# Effect of Organic Selenium-Enriched Yeast on Relieving the Deterioration of Layer Performance, Immune Function, and Physiological Indicators Induced by Heat Stress

**DOI:** 10.3389/fvets.2022.880790

**Published:** 2022-04-28

**Authors:** Ahmed O. Abbas, Abdulaziz A. Alaqil, Gamal M. K. Mehaisen, Mohamed I. El Sabry

**Affiliations:** ^1^Department of Animal and Fish Production, College of Agricultural and Food Sciences, King Faisal University, Al-Ahsa, Saudi Arabia; ^2^Department of Animal Production, Faculty of Agriculture, Cairo University, Giza, Egypt

**Keywords:** organic selenium, heat stress, productive performance, immunity, physiology, laying hens

## Abstract

Heat stress (HS) induces deleterious effects on the performance of laying hens and causes economic losses for poultry industry. This study was carried out to investigate the organic effect of selenium-enriched yeast (SY) on relieving the performance, immunity and physiological deterioration induced by heat stress in laying hens. A total of 324, 28-week-old, Hy-Line Brown commercial chicken layers were randomly distributed into 4 treatments according to a 2 × 2 factorial design, with 9 hens × 9 replicates per treatment (*n* = 81). From 30 to 34 weeks of age, layers were exposed to 2 temperature treatments (the HS treatment groups): a thermoneutral temperature at 24°C and a heat stress at 35°C. Layers were further assigned into the 2 subgroups according to dietary supplementation with organic selenium-enriched yeast (the SY treatment groups) at either 0 or 0.4 mg/kg diet. Results indicated that all the aspects of the layer performance during the experimental period were impaired by exposure to HS, while SY supplementation improved the layer performance in both the HS and non-HS layers. Intestinal villi disruptions and liver necrotic hepatocytes were observed in the layers exposed to HS, while villi integrity and hepatocytic normality were enhanced by SY treatment. A significant (*P* < 0.05) decrease in the total leukocyte count, sheep red blood cell (SRBC) antibody titer, and T- and B-lymphocyte proliferation along with an increase in the heterophils/lymphocytes (H/L) ratio were observed in the HS layers compared to non-HS layers. On the contrary, SY treatment significantly (*P* < 0.05) improved the immune function traits in both the HS layers and non-HS layers. Furthermore, the SY treatment plays an important role in mitigating the oxidative stress and inflammation induced by HS, displaying lower levels of plasma corticosterone, lipid peroxidation, interleukin-1β, and tumor necrosis factor-α in HS layers supplemented with SY compared to HS layers without SY supplementation. These results conclude that addition of SY to the diet of laying hens could be applied as a potential nutritional approach to relieve the deterioration effects of heat stress on the immunity, physiological status, and productive performance of laying hens.

## Introduction

The incidence of dramatically high heat waves, especially during the summer season in some desert and tropical areas, causes heat stress to poultry flocks. In commercial egg production, heat stress has deleterious effects on the performance of laying hens, starting from decreased feed intake, impaired gut formation, and reduced dietary digestibility, which, in turn, lead to a substantial decrease in body weight, egg production, feed efficiency, and egg quality (1–4). Heat stress also depresses the immune function of laying hens, including a general reduction in blood leukocytes, specific reduction in circulating lymphocytes in contrast with an increment in circulating heterophils, and the suppression of humoral and cell-mediated immune responses (3–6). Furthermore, it was reported that laying hens exposed to heat stress expressed an elevation in serum inflammatory cytokines, such as tumor necrosis factor-α, interleukin-1, and interlukin-6, accompanied with an elevation in the stress hormone, corticosterone (7, 8). The exposure to heat stress also initiated lipid peroxidation in bird cells as a harmful consequence of excess generation of reactive oxygen species (ROS) and free radicals (9). Birds under heat stress condition redirect their productive energy into intensive physiological procedures to maintain their homeostasis and livability and this, consequently, leads to a substantial economic losses in poultry production (10). The deterioration effect of heat stress on poultry production is widely discussed in the literatures; however, some of the biological mechanisms and physiological pathways that exert this effect still need deeply study.

Selenium (Se) is an important trace element that can prevent damage of organs under unfavorable conditions through its participation in carbohydrate, protein, and lipid metabolism (11) and its role in maintaining the antioxidant defense systems (12). It is well documented that Se serves as a cofactor for the function of antioxidant enzymes such as glutathione peroxidase (13). In addition, the role of Se incorporated in proteins is crucial for cellular processes involved in the cell-mediated and humoral immunity of poultry species (14). Se requirement for laying hens was reported in the range of 0.05–0.08 ppm (15) and its deficiency leads to disorder symptoms in chickens (16). Selenium-enriched yeast (SY) is a bioavailable abundant source of organic selenium in the form of selenomethionine (17). Compared with other inorganic salts of Se such as sodium selenite, the SY might be more safe, less toxicity, better absorption, and higher utilization by the laying hens (18–22).

There are few published studies with diverse results for the effects of selenium sources (inorganic vs. organic) and levels (ranged 0.12–0.4 mg/kg diet) on the egg quality and productive performance of laying hens (3, 4, 18, 22–26). In addition, the physiological mechanisms associated to the reported effects of organic selenium, particularly under heat stress conditions, remain unclear. Therefore, this study was conducted to illuminate the effect of yeast-organic selenium supplementation into laying hens' diets on minimizing the deterioration of layer performance, immunity, and physiological indicators induced by heat stress.

## Materials and Methods

### Ethical Standards

This study applied a heat stress protocol on layer chickens. To minimize suffering of birds during the experimental period, monitoring of birds was accomplished twice a day to detect any signs of chronic stress such as breathing difficulty, watery discharge of the peak, decreased appetite, ruffled feathers, or droopy looking. When one or more of such signs appeared, euthanasia was allowed by cervical dislocation to end the life of these birds. This study was approved by the Institutional Animal Care and Use Committee at Cairo University (CU-IACUC) reviewers and given the approval number CU-II-F-27-20.

### Birds and Management

A total of 324, 28-week-old, Hy-Line Brown commercial chicken layers were used in this study. The layers were distributed equally into two identical environmentally-controlled rooms (162 layers each room) and settled in individual standard wired cages (40 cm × 40 cm × 50 cm). Rooms were supplied with a constant light-emitting diode (LED) light of 17 h/day, light intensity of 30 Lux, temperature at 24°C, and 50% relative humidity. A basal diet was formulated to meet the Hy-Line Brown's nutritional recommendations at the production period (Table 1). The nutrients of the basal diet were calculated by using a classic reference for poultry nutrition (15) and the chemical analysis was determined as described by the Association of Official Analytical Chemists (AOAC) (27). Birds had free access to feed and water during the experimental period (30–34 weeks of age).

**Table 1:** Ingredient composition and chemical analysis of the basal diet.

**Ingredients**	**Content (g per kg diet)**
Yellow corn	565.5
Soybean meal (44%)	276.0
Wheat bran	10.0
Soybean oil	30.0
Bone meal	30.0
Limestone	80.0
Sodium chloride	4.0
DL-Methionine	1.5
Premix^a^	3.0
**Calculated nutrient analysis^b^**	**Value per kg diet**
Metabolizable energy (MJ)	1.26
Crude protein (g)	174.7
Calcium (g)	40.2
Available phosphorus (g)	5.2
Lysine (g)	9.5
Methionine (g)	4.2
**Determined nutrient analysis^c^**	**Value per kg diet**
Dry matter (g)	890.0
Total ash (g)	129.0
Crude protein (g)	167.5
Crude fat (g)	66.0
Crude fiber (g)	47.0
Calcium (g)	42.2
Available phosphorus (g)	4.2

a *Supplied the following vitamins and minerals: 8000 IU vitamin A; 1500 IU vitamin D; 4 mg riboflavin; 10 μg cobalamin; 15 mg vitamin E; 2 mg vitamin K; 500 mg choline; 25 mg niacin; 60 mg manganese; 50 mg zinc*.

b *Calculated by using the classic reference for poultry nutrition (15)*.

c *Analyzed according to AOAC (27)*.

### Experimental Design and Treatments

From 30 to 34 weeks of age, layers were randomly allotted to 4 treatments according to a 2 × 2 factorial design, with nine replicates containing nine birds per treatment (*n* = 81). Layers in the first room were kept in a thermoneutral temperature at 24°C and those in the other room were exposed to heat stress at 35°C (the HS treatment groups). Birds in each room were further assigned into the two equal subgroups according to dietary supplementation with organic selenium-enriched yeast (Se-yeast Sel-Plex® Alltech, Nicholasville, USA) at either 0 or 0.4 mg/kg diet (the SY treatment groups). The total Se contents were analyzed in the basal diets with or without SY (triplicate determination each) and the values obtained were 0.446 and 0.045 mg/kg, respectively. The productive performance traits were recorded during the experimental period as mentioned later. Blood samples were obtained to analyze the immune function and physiological stress indicators of layers. In addition, small intestines and liver specimens were harvested from layers to evaluate the histomorphological changes between the treatment groups.

### Productive Performance

Total egg number (EN), average egg weight (EW), and daily feed intake (FI) were recorded per hen during the experimental period (30–34 weeks of age). The total feed consumption and egg mass (EN × EW) were calculated per hen during the whole experimental period. Feed conversion ratio (FCR) was also calculated per hen on the basis of total feed consumption/total egg mass per hen during the whole experimental period. Egg and feed weights were obtained by using LCD digital balance of 10 kg maximum weight and 0.5 g accuracy (DT580, Atrontec Electronic Tech Corporation Ltd., China).

### Immune Function

#### Leukocytes Count and Differentiation

At the end of the experimental period (34 weeks of age), blood samples were withdrawn from the wing vein of 9 layers per treatment subgroup and transferred into heparinized tubes. 10 μl of the blood sample was diluted with brilliant cresyl blue stain solution (1:50 v/v) and a drop of mixture was mounted on a hemocytometer slide. The total leukocytes (TLs) were then counted under a microscope at a magnification of 200X (28). Another 10 μl of the blood sample was smeared on a glass slide, fixed with methyl alcohol, and then stained using Hema 3 Solution (Thermo Fisher Scientific, Pittsburg, Pennsylvania, USA). Approximately, 200 leukocytes were differentiated under a microscope at a magnification of 1,000X with oil immersion. The heterophil to lymphocyte (H/L) ratio was then calculated (29).

#### Sheep Red Blood Cells Antibody Titer

The sheep red blood cells antibody (SRBC-AB) titer of layers was detected according to methods described in a previous study (30). In brief, 9 layers from each treatment subgroup were intravenously injected with 1 ml of 5% SRBC 1 week before the end of the experiment. After that, blood samples were collected from the layers and centrifuged at 220 × *g* at room temperature and the sera were then separated and stored at −20°C. Serial doubling dilutions of sera samples (25 μl each) were pipetted in a 96-well plate and 25 μl of 2% SRBC solution was put on each dilution. The plates were gently vortexed for few seconds and then left overnight for agglutination at room temperature. SRBC-AB titer was expressed as log_2_ of the inverse of the last dilution exhibiting positive agglutination in the bottom of the well.

#### T- and B-Lymphocyte Proliferation

Nine blood samples were obtained from different layers in each treatment subgroup to evaluate T- and B-cells lymphocyte proliferation according to methods recently described in a previous study (31). First, the blood sample was overlayed on the top of an equivalent volume of Histopaque-1077 separation medium (Sigma Chemical Corporation, St. Louis, Mosby, USA) in a falcon tube and centrifuged at 1,030 × g for 20 min at 4°C. The isolated peripheral blood mononuclear cells (PBMCs) as a layer on the histopaque interface were carefully aspirated and washed twice with RPMI-1640 medium (Invitrogen Corporation, Grand Island, New York, USA) and then resuspended in 1 ml of RPMI-1640 medium. The concentration of viable lymphocytes in each sample was detected by Trypan Blue dye and then readjusted at 10 million cells/ml in triplicates in 96-well plates. Experimental wells were supplemented with 50 μl of either 5% concanavalin A mitogen or 1% lipopolysaccharide to induce T- or B-lymphocyte proliferation, respectively, while control wells were supplemented only with 50 μl RPMI-1640 medium. The plates were incubated at 42°C, 5% CO_2_, and saturated humidity for 48 h; then, 15 μl of 3-(4, 5-Dimethylthiazol-2-yl)-2,5-diphenyltetrazolium bromide (MTT) (0.5%) was added to each well and further incubated for 4 h. Finally, 100 μl solution of 10% sodium dodecyl sulfates in 0.04 M HCl was added to each well and then the optical density at 570 nm (OD570) was recorded for the experimental against control wells using an automated ELISA (Bio-Rad Laboratories Incorporation, USA). Stimulating indexes for T- and B-lymphocytes (TSI and BSI, respectively) were calculated as the ratio of OD570 for stimulated to unstimulated cells in each sample.

### Physiological Stress Indicators

Blood samples were collected into heparinized tubes from 9 layers in each treatment subgroup at the end of the experimental period (34 weeks of age). Plasma was separated by centrifugation of blood samples at 2,000 × g for 10 min at 4°C and stored at −20°C until further analysis of physiological stress indicators. Plasma interleukin-1β (IL-1β), tumor necrosis factor-α (TNF-α), and corticosterone (CORT) concentrations were assayed according to the manufacturer protocols of chicken ELISA kits (MyBioSource Incorporation, San Diego, California, USA; Cat no. MBS761055, MBS2509660, and MBS701668, respectively). The intra- and interassay CV% were <10 and <12% for IL-1β, <5.57 and <5.89% for TNF-α, and <8 and <10% for CORT, respectively. The sensitivities and detection ranges were 0.02 and 0.03–2 ng/ml, 18.75 and 31.25–2,000 pg/ml, and <0.5 and 0.5–20 ng/ml for IL-1β, TNF-α, and CORT, respectively. Furthermore, the lipid peroxidation product [malondialdehyde (MDA)] was estimated in the plasma by using the Colorimetric Assay Kit (MyBioSource Incorporation, San Diego, California, USA; Cat no. MBS9718963). According to the manufacturer's guideline, 100 μl of the plasma was mixed with 300 μl of thiobarbituric acid (TBA) solution and incubated for 30 min at 95°C. After cooling in an ice bath for 10 min, the mixture was centrifuged at 10,000 × g for 10 min at 25°C. Aliquots of 200 μl of the supernatants were then transferred to 96-well microplate and the absorbance was obtained at 532 nm using a microplate reader (ELx808™ BioTek Instruments, Winooski, Vermont, USA).

### Intestinal and Liver Histomorphology

At the end of experiment, 9 layers from each treatment subgroup were decapitated and the representative specimens of liver and small intestine (jejunum) were separated from the rest of the gastrointestinal tract. Liver and intestine specimens were fixed in 10% formalin-saline solution and then processed into sections using a rotatory microtome and the ordinary paraffin embedding technique. At least, 5 cross-sections per sample (3–5 μm in thickness) were stained with H&E as outlined in a study by Suvarna et al. (32). The histomorphological changes of liver and intestinal sections were observed and photographed using light microscope (400X magnification) connected with HD digital camera (Leica Microsystems, Germany). The intestinal villus height (VH), crypt depth (CD), and VH/CD ratio were also recorded for each treatment subgroup.

### Statistical Analysis

Data were analyzed by a two-way ANOVA using general linear model (SPSS 2013, Software Package version 22.0, IBM Corporation, New York, USA). The main effects were heat stress (HS) (24 vs. 35°C) and dietary organic selenium supplementation (SY) (0 vs. 0.4 mg/kg). The interaction between the two main factors (HS × SY) was tested. The experimental unit was considered as the number of observations per group for each test done (*n* = 81 for productive performance traits, while *n* = 9 for the data of immune function, physiological stress indicators, and intestinal histomorphology). Mean differences were tested at 0.05 level of significance using the Tukey's honestly significant difference (HSD) test.

## Results

### Productive Performance

The results of productive performance traits as affected by HS, SY, and their interaction (HS × SY) are shown in Table 2. The results indicated that exposure to HS significantly (*P* < 0.05) decreased the EN, EW, and FI and impaired the FCR. In contrast, SY supplementation into layer diets significantly (*P* < 0.05) improved all the aspects of the layer performance. Moreover, a significant interaction effect was exerted on EN, FI, and FCR (*P* < 0.05), but no interaction effect was exerted on the EW. When SY was supplemented to the diets of HS layers, the reduction in EN, FI, and FCR was significantly (*P* < 0.05) ameliorated, but still lower than that in layers exposed to thermoneutral condition.

**Table 2:** Effect of dietary organic selenium supplementation on the productive performance of laying hens under heat stress condition.

		**Traits** ^**1**^			
**Treatment groups** ^**2**^		**EN**	**EW (g)**	**FI (g)**	**FCR**
HS (°C)	24	26.59^a^	60.25^a^	114.96^a^	2.01^b^
	35	20.64^b^	53.43^b^	108.19^b^	2.78^a^
	SEM	0.089	0.092	0.131	0.012
SY (mg/kg)	0	22.55^b^	55.73^b^	110.41^b^	2.55^a^
	0.4	24.68^a^	57.95^a^	112.74^a^	2.25^b^
	SEM	0.089	0.092	0.131	0.012
HS × SY_(n)_	24 × 0	26.06^b^	59.22	114.07^b^	2.07^c^
	35 × 0	19.04^d^	52.24	106.74^d^	3.02^a^
	35 × 0.4	22.24^c^	54.62	109.64^c^	2.54^b^
	24 × 0.4	27.12^a^	61.28	115.84^a^	1.95^d^
	SEM	0.127	0.130	0.186	0.017
*P*-value	HS	<0.001	<0.001	<0.001	<0.001
	SY	<0.001	<0.001	<0.001	<0.001
	HS × SY	<0.001	0.219	0.002	<0.001

*Means with different superscripts in the same column indicate significant differences (P < 0.05), whereas means with the same or no superscripts indicate no significant differences (P > 0.05)*.

1 *Traits: EN, egg number per hen during the experimental period (30–34 wk of age); EW, average egg weight; FI, feed intake per hen per day; FCR, feed conversion ratio calculated as kg feed/ kg egg mass*.

2 *Treatment groups: HS, layers were exposed to either thermoneutral temperature at 24°C or heat stress at 35°C; SY, layers were fed basal diet containing 0 or 0.4 mg/kg selenium-enriched yeast; HS × SY, interaction between HS and SY groups (n = 81 layers per subgroup). SEM, standard error of the mean*.

### Immune Function

The results in Table 3 display the effect of HS, SY, and their interaction (HS × SY) on the immune function traits. A significant decrease in the total leukocyte (TL) count, SRBC-AB titer, TSI and BSI, and an increase in the H/L ratio were observed in HS layers compared to non-HS layers (*P* < 0.05). On the contrary, a significant decrease in the H/L ratio and significant increase in the TL count, SRBC-AB titer, TSI, and BSI were noticed in the SY layers group compared to the non-SY layers group (*P* < 0.05). There was a significant (*P* < 0.05) interaction effect between HS and SY treatments for all the immunological traits. The SY treatment significantly improved the immune function traits in the HS layers compared to nontreated control layers.

**Table 3:** Effect of dietary organic selenium supplementation on the immune function of laying hens under heat stress condition.

		**Traits** ^**1**^				
**Treatment groups** ^**2**^		**TL count (10^**3**^/mL)**	**H/L ratio**	**SRBC-AB titer (log_**2**_)**	**TSI**	**BSI**
HS (°C)	24	61.27^a^	0.34^b^	8.61^a^	3.08^a^	2.31^a^
	35	40.34^b^	0.78^a^	5.61^b^	1.79^b^	1.68^b^
	SEM	1.288	0.019	0.212	0.080	0.052
SY (mg/kg)	0	47.30^b^	0.62^a^	6.39^b^	2.04^b^	1.56^b^
	0.4	54.31^a^	0.50^b^	7.83^a^	2.83^a^	2.43^a^
	SEM	1.269	0.019	0.212	0.080	0.052
HS × SY_(n)_	24 × 0	59.63^a^	0.34^c^	8.22^a^	2.81^b^	1.96^b^
	35 × 0	34.97^c^	0.89^a^	4.56^c^	1.26^d^	1.16^c^
	35 × 0.4	45.72^b^	0.67 ^b^	6.67^b^	2.31^c^	2.21^b^
	24 × 0.4	62.90^a^	0.33^c^	9.00^a^	3.34^a^	2.66^a^
	SEM	1.795	0.027	0.299	0.128	0.073
*P*-value	HS	<0.001	<0.001	<0.001	<0.001	<0.001
	SY	<0.001	<0.001	<0.001	<0.001	<0.001
	HS × SY	0.045	0.001	0.033	0.028	0.024

*Means with different superscripts in the same column indicate significant differences (P < 0.05), whereas means with the same or no superscripts indicate no significant differences (P > 0.05)*.

1 *Traits: TL count, total leukocytes count; H/L ratio, heterophil to lymphocyte cells ratio; SRBC-AB titer, sheep red blood cells antibody titer; TSI, T-lymphocyte stimulation index; BSI, B-lymphocyte stimulation index*.

2 *Treatment groups: HS, layers were exposed to either thermoneutral temperature at 24°C or heat stress at 35°C; SY, layers were fed basal diet containing 0 or 0.4 mg/kg selenium-enriched yeast; HS × SY, interaction between HS and SY groups (n = 9 samples per subgroup). SEM, standard error of the mean*.

### Physiological Stress Indicators

The results in Table 4 show significant effects (*P* < 0.05) for HS, SY, and their interaction on all the physiological stress indicators examined in this study. The plasma IL-1β, TNF-α, CORT, and MDA were significantly (*P* < 0.05) increased due to exposure of layers to HS. On contrary, these stress indicators were significantly (*P* < 0.05) decreased due to SY supplementation into layer diets. Furthermore, SY treatment significantly (*P* < 0.05) reduced the elevation of IL-1β, TNF-α, CORT, and MDA in the layers exposed to HS.

**Table 4:** Effect of dietary organic selenium supplementation on the physiological stress indicators of laying hens under heat stress condition.

		**Traits** ^**1**^			
**Treatment groups** ^**2**^		**IL-1β (ng/mL)**	**TNFα (pg/mL)**	**CORT (pg/mL)**	**MDA (μM/mL)**
HS (°C)	24	0.26^b^	89.47^b^	4.61^b^	1.91^b^
	35	0.67^a^	162.70^a^	11.34^a^	3.63^a^
	SEM	0.021	2.943	0.380	0.126
SY (mg/kg)	0	0.56^a^	135.77^a^	9.34^a^	3.43^a^
	0.4	0.37^b^	116.40^b^	6.61^b^	2.12^b^
	SEM	0.021	2.943	0.380	0.126
HS × SY_(n)_	24 × 0	0.28^c^	91.43^c^	4.91^c^	2.16^bc^
	35 × 0	0.83^a^	180.12^a^	13.78^a^	4.70^a^
	35 × 0.4	0.50^b^	145.28^b^	8.90^b^	2.56^b^
	24 × 0.4	0.24^c^	87.52^c^	4.32^c^	1.67^c^
	SEM	0.029	4.162	0.537	0.179
*P*-value	HS	<0.001	<0.001	<0.001	<0.001
	SY	<0.001	<0.001	<0.001	<0.001
	HS × SY	<0.001	0.001	<0.001	<0.001

*Means with different superscripts in the same column indicate significant differences (P < 0.05), whereas means with the same or no superscripts indicate no significant differences (P > 0.05)*.

1 *Traits: IL-1β, interleukin 1 beta; TNFα, tumor necrosis factor alpha; CORT, corticosterone; MDA, malondialdehyde*.

2 *Treatment groups: HS, layers were exposed to either thermoneutral temperature at 24°C or heat stress at 35°C; SY, layers were fed basal diet containing 0 or 0.4 mg/kg selenium-enriched yeast; HS × SY, interaction between HS and SY groups (n = 9 samples per subgroup). SEM, standard error of the mean*.

### Intestinal and Liver Histology

**Figure 1:**
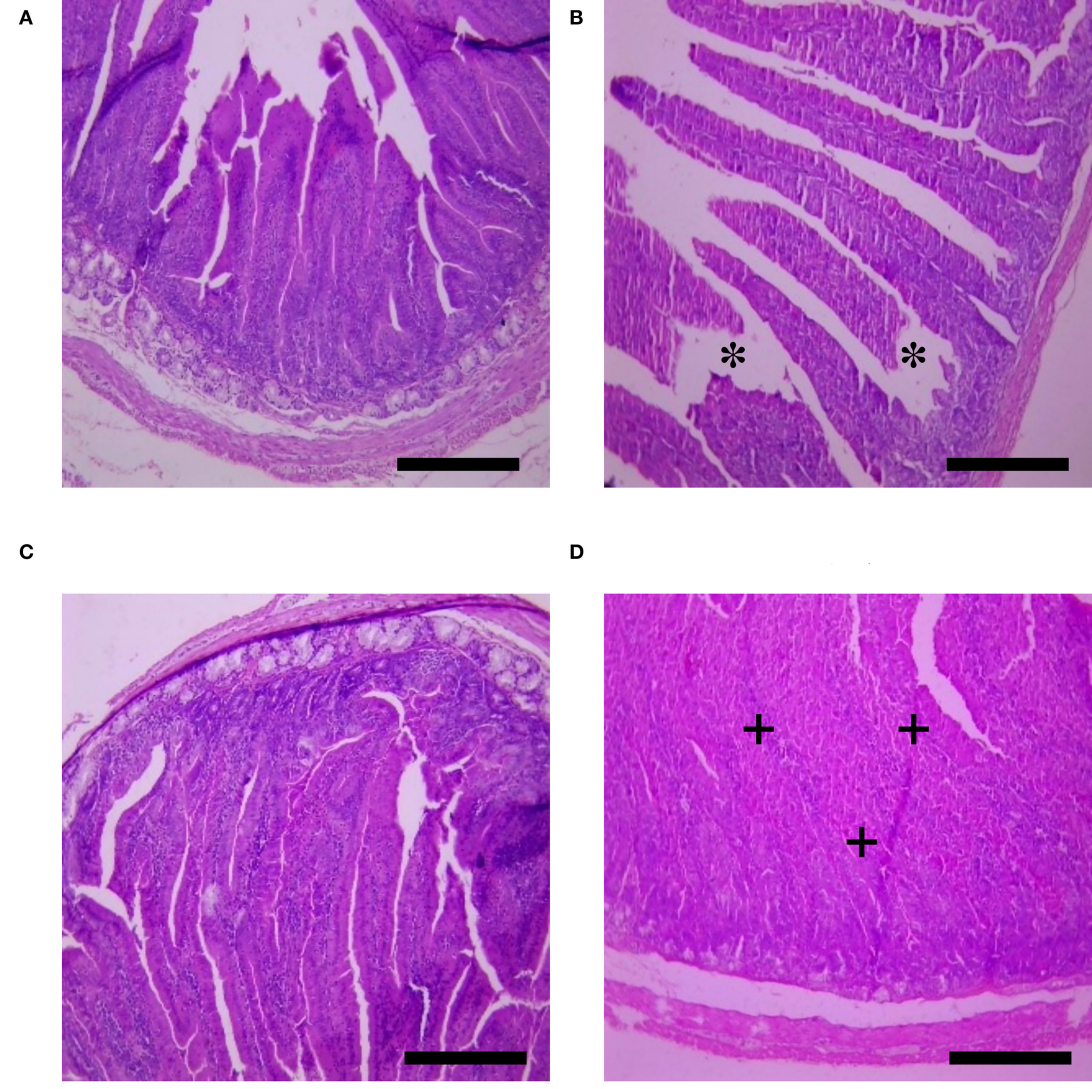
Histological sections of small intestines of layers exposed to heat stress (HS) and supplemented with organic selenium-enriched yeast (SY) in the basal diet (scale bars 25 μm). **(A)** Layers exposed to thermoneutral temperature at 24°C and fed a basal diet without SY supplementation; **(B)** Layers exposed to HS at 35°C and fed a basal diet without SY supplementation; **(C)** Layers exposed to HS at 35°C and fed a basal diet supplemented with 0.4 g/kg SY; **(D)** Layers exposed to thermoneutral temperature at 24°C and fed a basal diet supplemented with 0.4 g/kg SY. Asterisks (*) indicate the disruption of intestinal villi, while plus signs (+) indicate the integrity of intestinal villi.

**Figure 2:**
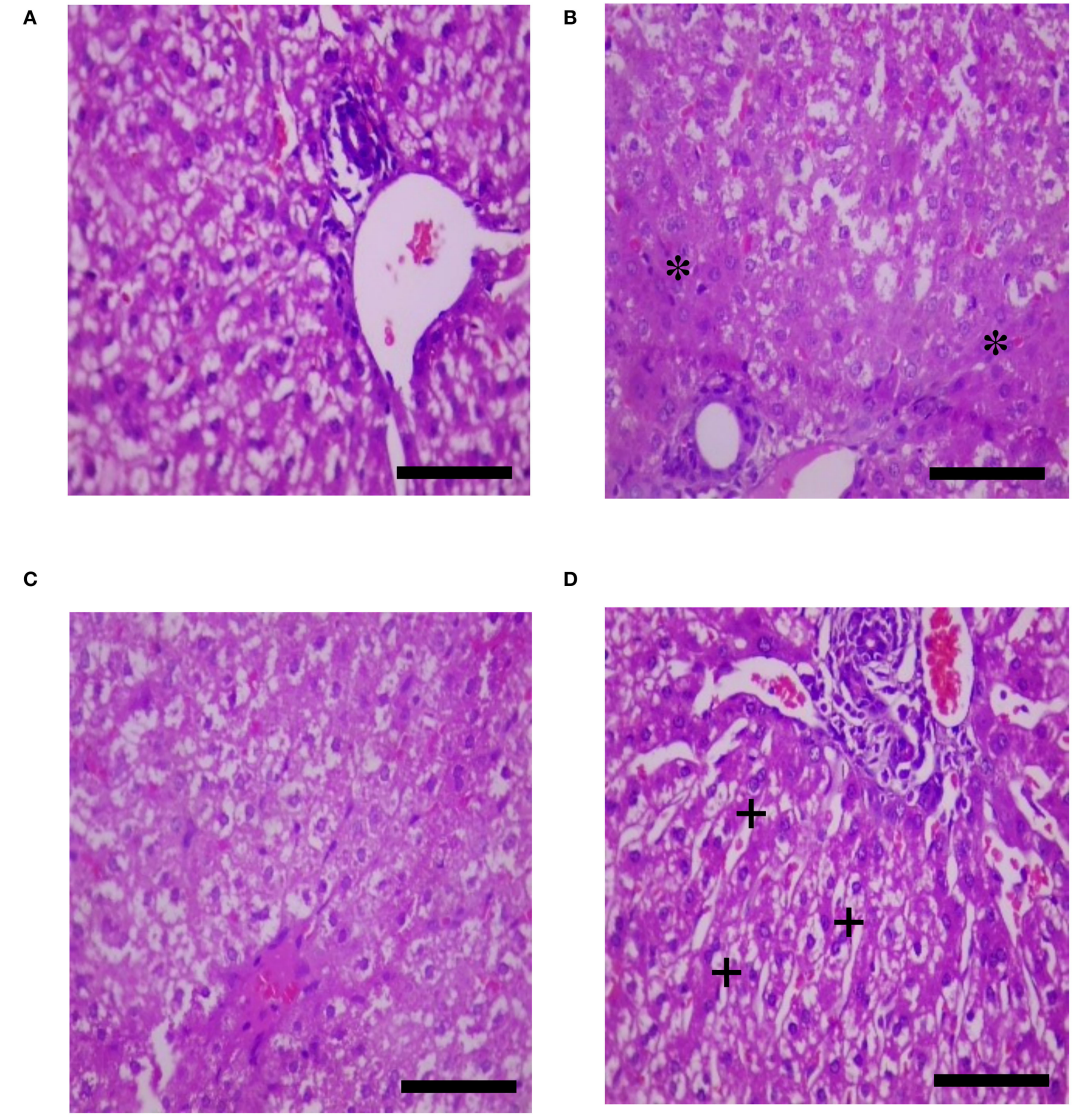
Histological sections of livers of layers exposed to heat stress (HS) and supplemented with organic selenium-enriched yeast (SY) in the basal diet (scale bars 25 μm). **(A)** Layers exposed to thermoneutral temperature at 24°C and fed a basal diet without SY supplementation; **(B)** Layers exposed to HS at 35°C and fed a basal diet without SY supplementation; **(C)** Layers exposed to HS at 35°C and fed a basal diet supplemented with 0.4 g/kg SY; **(D)** Layers exposed to thermoneutral temperature at 24°C and fed a basal diet supplemented with 0.4 g/kg SY. Asterisks (*) indicate the areas of hepatocytic necrosis, while plus signs (+) indicate the normal hepatocytic acini.

The effects of HS, SY, and HS × SY interaction on the intestinal histomorphology of layers are given in Table 5. No interaction effects were obtained between HS and SY treatments on the histomorphological measurements of layer intestines. The VH and CD of layer intestines were significantly declined by HS, while they were enhanced by SY treatment (*P* < 0.05). The microscopic examination of the intestinal sections obtained from the different groups is shown in Figure 1. In addition to the VH and CD declines, clear villi disruptions were also observed in the layers exposed to HS (Figure 1B) compared to those exposed to thermoneutral conditions (Figure 1A), while enhanced villi integrity and coherence were observed in addition to the increased VH and CD in the layers fed with SY (Figure 1D) compared to those layers fed without SY (Figure 1A). On the other hand, the histology of layer livers from all the treatment groups is given in Figure 2. Liver sections of the HS layers group (Figure 2B) displayed necrotic areas of hepatocytes, while those liver sections from the SY layers group (Figure 2D) displayed more normal hepatocytic acini compared to the layers in thermoneutral condition without SY supplementation (Figure 2A).

**Table 5:** Effect of dietary organic selenium supplementation on the intestinal histomorphology of laying hens under heat stress condition.

		**Traits** ^**1**^		
**Treatment groups** ^**2**^		**VH (μm)**	**CD (μm)**	**VH/CD ratio**
HS (°C)	24	2,053.17^a^	447.17^a^	4.63
	35	1,845.17^b^	382.50^b^	4.85
	SEM	29.570	8.941	0.120
SY (mg/kg)	0	1,857.39^b^	394.28^b^	4.76
	0.4	2,040.94^a^	435.39^a^	4.71
	SEM	29.570	8.941	0.120
HS × SY_(n)_	24 × 0	1,942.11	422.56	4.63
	35 × 0	1,772.67	366.00	4.89
	35 × 0.4	1,917.67	399.00	4.81
	24 × 0.4	2,164.22	471.78	4.62
	SEM	41.818	12.645	0.170
*P*-value	HS	<0.001	<0.001	0.204
	SY	<0.001	0.003	0.803
	HS × SY	0.363	0.526	0.828

*Means with different superscripts in the same column indicate significant differences (P < 0.05), whereas means with the same or no superscripts indicate no significant differences (P > 0.05)*.

1 *Traits: VH, villus height; CD, Crypt depth; VH/CD ratio, villus height to crypt depth ratio*.

2 *Treatment groups: HS, layers were exposed to either thermoneutral temperature at 24°C or heat stress at 35°C; SY, layers were fed basal diet containing 0 or 0.4 mg/kg selenium-enriched yeast; HS × SY, interaction between HS and SY groups (n = 9 samples per subgroup). SEM, standard error of the mean*.

## Discussion

The deterioration effects of heat stress on the productive performance of laying hens were evidenced in this study. In consistence with other previous study (1, 2, 33, 34), a substantial depression was noticed in the EN and EW for HS layers when compared to its control. The obvious decrease in egg quantity could be reasoned directly to the depression in FI and FCR of the same group of HS birds (3, 4, 35). However, other physiological alterations happened in HS layers and might contribute to the low performance of laying hens in this study. HS resulted in intestinal villi disruption and hepatocytic necrosis (Figures 1, 2). Similar damages were also observed in the intestinal morphology of quail birds (36), broilers (10), laying hens (33), and in broiler livers (37) when exposed to HS. Such morphological damages normally result in poor nutrient digestion and absorption (38) and, consequently, lead to low animal performance (39).

Animal and poultry studies have evidenced an extent association between stress factors and immune dysregulation (40). Our results agree with this finding that layers exposed to HS expressed a significant suppression in all the immunological measurements (Table 3) along with an elevation in the plasma stress indicators (Table 4). HS is known to limit the immunocompetence of laying hens through decreasing the humoral immunity (41), as indicated herein by the decrease in TL count and anti-SRBC-AB titer. It is well described that HS activates the hypothalamic-pituitary-adrenal (HPA) axis followed by an elevation in the secretion of stress hormone, CORT (42). The results of this study and that of previous studies (43–46) indicated that increased plasma CORT in the HS layers led to an elevation in the H/L ratio and an inhibition in the lymphocyte proliferation (TSI and BSI). The increased H/L ratio in the HS layer group could be attributed to endotoxins and heterophils infiltration from damaged mucosal intestines in these birds (47). Furthermore, HS increased other plasma stress indicators assessed in this study, including the proinflammatory cytokines (TNF-α and IL-1β) and lipid peroxidation (MDA). The increase in TNF-α may be due to the secretion of CORT (48). It was recently reported that HS impaired egg production by inducing follicular cells apoptosis through producing ROS/MDA and activating the CORT/TNF-α pathways (49).

In the recent years, our previous studies have focused on understanding the physiological mechanisms behind the negative effects of heat stress on poultry production (43, 45) and suggested nutritional strategies to minimize the deterioration induced by HS in different poultry species (36, 44, 50). In this trend, SY was supplemented into diets of laying hens and considerable results were obtained in this study. The productive performance traits were significantly higher in the layers fed with SY than those fed without SY. Similar results were obtained in laying hens (18, 51, 52) and in quail birds (53). In other studies, no effects for Se inclusion into diets were found on the laying performance (20, 23, 54). The variation in Se effects on performance may be correlated to poultry species, age, ration composition, environmental condition, and Se sources and levels itself (20). In this study, SY supplementation to HS layers relieved the low performance induced by HS in the EN, FI, and FCR. In line with these results, Sahin et al. (55) found that egg production, egg quality, feed intake, and feed efficiency were significantly improved by adding SY at 0.3 mg/kg to the diets of HS-quail birds. It was reported that SY is absorbed in the small intestinal mucosa as intact molecules through a specific pathway of methionine (56). The absorbed Se then incorporates fundamentally in the functions of some metabolic enzymes, such as iodothyronine deiodinases, which produce active thyroid hormones and redox enzymes, such as thioredoxin reductase and glutathione peroxidase, which reduce accumulation of hydrogen peroxides and consecutive oxidative damage (12). These functions may be the main contribution to enhance and/or maintain the membrane integrity of vital tissues (57), as shown in this histological study by the obvious integrity of intestinal villi and normal hepatocytic acini in the layers received SY than the other groups. In addition, Se is crucial for adequate appetite, efficient feed utilization, and active metabolism of carbohydrates, lipids, and proteins (11), thereby improve the other performance aspects (58).

On the other hand, current results showed that dietary SY supplementation had positive effects on the immune function of laying hens. It was well documented that dietary Se, especially in the form of selenoproteins, has an important role in regulating the immune responses in poultry (14). The increased TL count and decreased H/L ratio in the SY groups vs. the non-SY groups may be due to the coherence and integrity of small intestine villi and, thus, prevent heterophils infiltration as mentioned before (47). The lower H/L ratio in SY layers also indicated that SY treatment was able to lower the stress status of laying hens (59). In contrast, SY treatment in this study activated both the humoral immunity via increasing SRBC-AB and the cell-mediated immunity via increasing TSI and BSI. Similar beneficial effects of dietary supplementation with organic sources of Se on the humoral and cellular immune responses were previously reported in chickens (60–62) and quail birds (63–65). This increase in the lymphocyte proliferation by SY could be interpreted by previous reports that Se may augment the expression of IL-2 receptors on T cells, increasing T- and B-lymphocyte cells proliferation and responses (66).

Besides the decrease in H/L ratio, other stress indicators were reduced by SY supplementation to the diets of layers exposed to either neutral temperatures or heat stress (Table 4). Some previous studies (67–69) and the present experiment showed that dietary Se supplementation could improve antioxidative status of chickens under heat stress condition. SY supplementation to HS-layer diets decreased the plasma MDA levels because SY can activate the biological activity of antioxidant enzymes, especially glutathione peroxidases, which, in turn, prevent the harmful effect of hydrogen peroxides and reduce the formation of reactive oxidative products (12, 70). When layers were exposed to HS, SY supplementation reduced the levels of proinflammatory cytokines IL-1β and TNF-α through inhibiting the nuclear factor kappa-B (NF-κB) signaling pathway (71).

## Conclusion

The results of this study indicate that dietary organic SY supplementation at 0.4 mg/kg can relieve the deleterious effects of heat stress on the productive performance and immune function of laying hens. The SY treatment plays an important role in mitigating the oxidative stress and inflammation induced by HS, displaying lower levels of plasma CORT, H/L ratio, MDA, IL-1β, and TNF-α. The total leukocytes, antibody production, and lymphocyte proliferation were also improved by the addition of SY into layer diets, particularly during heat stress. In addition, the SY treatment preserved the integrity of intestinal villi and the normality of liver hepatocytic acini, which consequently enhanced the feed intake and efficiency and the other productive performance of laying hens. Therefore, the addition of SY to the diet of laying hens could be recommended as a potential nutritional strategy in order to improve the performance, especially under heat stress conditions.

## Data Availability Statement

The original contributions presented in the study are included in the article/Supplementary Material, further inquiries can be directed to the corresponding authors.

## Ethics Statement

The animal study was reviewed and approved by the Institutional Animal Care and Use Committee at Cairo University (CU-IACUC). Written informed consent was obtained from the owners for the participation of their animals in this study (Approval code: CU-II-F-27-20).

## Author Contributions

AAl, AAb, ME, and GM: conceptualization, methodology, investigation, supervision, and project administration. AAb and GM: formal analysis and writing—review and editing. AAl and AAb: data curation. AAb, ME, and GM: writing—original draft preparation. All the authors agree to be accountable for the content of the manuscript. All authors contributed to the article and approved the submitted version.

## Funding

This study was supported through the annual funding track by the Deanship of Scientific Research, Vice Presidency for Graduate Studies and Scientific Research, King Faisal University, Saudi Arabia (Project No. AN000436).

## Conflict of Interest

The authors declare that the research was conducted in the absence of any commercial or financial relationships that could be construed as a potential conflict of interest.

## Publisher's Note

All claims expressed in this article are solely those of the authors and do not necessarily represent those of their affiliated organizations, or those of the publisher, the editors and the reviewers. Any product that may be evaluated in this article, or claim that may be made by its manufacturer, is not guaranteed or endorsed by the publisher.
