# Supplementary material for: Effect of Organic Selenium-Enriched Yeast on Relieving the Deterioration of Layer Performance, Immune Function, and Physiological Indicators Induced by Heat Stress
Source: Front Vet Sci. 2022 Apr 28;9:880790. doi: 10.3389/fvets.2022.880790 (PMC9096893; doi:10.3389/fvets.2022.880790)
Supplement: Supplementary file 1 [file Data_Sheet_1.pdf]

NO	REPLCT	CODE	GROUP	HS	SE	EN	EW	FI
1	1	CONT	0	0	0	26	59	113
2	1	CONT	0	0	0	27	60	111
3	1	CONT	0	0	0	25	60	115
4	1	CONT	0	0	0	26	59	115
5	1	CONT	0	0	0	26	58	112
6	1	CONT	0	0	0	27	58	114
7	1	CONT	0	0	0	28	59	111
8	1	CONT	0	0	0	25	60	116
9	1	CONT	0	0	0	26	60	115
10	2	CONT	0	0	0	25	59	114
11	2	CONT	0	0	0	25	59	114
12	2	CONT	0	0	0	26	60	115
13	2	CONT	0	0	0	27	58	114
14	2	CONT	0	0	0	26	58	114
15	2	CONT	0	0	0	25	59	115
16	2	CONT	0	0	0	25	58	113
17	2	CONT	0	0	0	26	60	114
18	2	CONT	0	0	0	25	60	113
19	3	CONT	0	0	0	26	58	116
20	3	CONT	0	0	0	26	60	112
21	3	CONT	0	0	0	26	59	112
22	3	CONT	0	0	0	25	59	114
23	3	CONT	0	0	0	25	58	114
24	3	CONT	0	0	0	28	60	114
25	3	CONT	0	0	0	28	59	115
26	3	CONT	0	0	0	25	60	112
27	3	CONT	0	0	0	26	60	111
28	4	CONT	0	0	0	27	60	113
29	4	CONT	0	0	0	27	59	116
30	4	CONT	0	0	0	26	59	113
31	4	CONT	0	0	0	26	60	114
32	4	CONT	0	0	0	26	60	115
33	4	CONT	0	0	0	28	58	115
34	4	CONT	0	0	0	27	60	112
35	4	CONT	0	0	0	27	59	114
36	4	CONT	0	0	0	26	59	113
37	5	CONT	0	0	0	26	60	113
38	5	CONT	0	0	0	26	58	115
39	5	CONT	0	0	0	25	58	112
40	5	CONT	0	0	0	26	59	114
41	5	CONT	0	0	0	27	58	114
42	5	CONT	0	0	0	26	60	116
43	5	CONT	0	0	0	26	60	113
44	5	CONT	0	0	0	25	59	114
45	5	CONT	0	0	0	25	60	115
46	6	CONT	0	0	0	26	60	113

47	6	CONT	0	0	0	26	59	116
48	6	CONT	0	0	0	25	58	114
49	6	CONT	0	0	0	27	59	112
50	6	CONT	0	0	0	27	59	115
51	6	CONT	0	0	0	26	60	112
52	6	CONT	0	0	0	25	58	113
53	6	CONT	0	0	0	26	60	114
54	6	CONT	0	0	0	25	60	113
55	7	CONT	0	0	0	26	60	114
56	7	CONT	0	0	0	26	59	116
57	7	CONT	0	0	0	26	60	112
58	7	CONT	0	0	0	26	58	115
59	7	CONT	0	0	0	26	58	116
60	7	CONT	0	0	0	25	59	113
61	7	CONT	0	0	0	27	60	112
62	7	CONT	0	0	0	25	60	114
63	7	CONT	0	0	0	26	60	114
64	8	CONT	0	0	0	26	59	112
65	8	CONT	0	0	0	26	60	115
66	8	CONT	0	0	0	25	59	112
67	8	CONT	0	0	0	26	59	116
68	8	CONT	0	0	0	27	60	114
69	8	CONT	0	0	0	28	58	114
70	8	CONT	0	0	0	25	60	113
71	8	CONT	0	0	0	27	60	114
72	8	CONT	0	0	0	26	59	118
73	9	CONT	0	0	0	26	59	114
74	9	CONT	0	0	0	25	60	116
75	9	CONT	0	0	0	27	60	116
76	9	CONT	0	0	0	27	58	114
77	9	CONT	0	0	0	27	60	117
78	9	CONT	0	0	0	26	59	118
79	9	CONT	0	0	0	26	59	117
80	9	CONT	0	0	0	25	58	117
81	9	CONT	0	0	0	27	60	116
82	1	HS	1	1	0	19	51	106
83	1	HS	1	1	0	20	55	110
84	1	HS	1	1	0	19	52	109
85	1	HS	1	1	0	21	55	107
86	1	HS	1	1	0	18	51	105
87	1	HS	1	1	0	18	52	104
88	1	HS	1	1	0	19	52	106
89	1	HS	1	1	0	19	53	107
90	1	HS	1	1	0	20	55	106
91	2	HS	1	1	0	20	52	106
92	2	HS	1	1	0	19	54	110
93	2	HS	1	1	0	19	52	109

94	2	HS	1	1	0	18	51	105
95	2	HS	1	1	0	21	52	106
96	2	HS	1	1	0	20	53	107
97	2	HS	1	1	0	18	52	107
98	2	HS	1	1	0	19	51	105
99	2	HS	1	1	0	17	53	108
100	3	HS	1	1	0	21	52	107
101	3	HS	1	1	0	20	52	106
102	3	HS	1	1	0	18	53	106
103	3	HS	1	1	0	19	51	110
104	3	HS	1	1	0	17	54	108
105	3	HS	1	1	0	18	52	110
106	3	HS	1	1	0	19	52	106
107	3	HS	1	1	0	20	52	105
108	3	HS	1	1	0	21	53	104
109	4	HS	1	1	0	20	51	107
110	4	HS	1	1	0	20	52	107
111	4	HS	1	1	0	20	51	106
112	4	HS	1	1	0	21	51	105
113	4	HS	1	1	0	18	54	108
114	4	HS	1	1	0	19	53	107
115	4	HS	1	1	0	17	52	105
116	4	HS	1	1	0	20	52	110
117	4	HS	1	1	0	19	51	109
118	5	HS	1	1	0	18	51	107
119	5	HS	1	1	0	19	51	106
120	5	HS	1	1	0	18	54	104
121	5	HS	1	1	0	20	52	104
122	5	HS	1	1	0	21	52	107
123	5	HS	1	1	0	19	53	108
124	5	HS	1	1	0	19	53	106
125	5	HS	1	1	0	18	53	109
126	5	HS	1	1	0	19	51	105
127	6	HS	1	1	0	19	53	104
128	6	HS	1	1	0	20	52	106
129	6	HS	1	1	0	17	52	104
130	6	HS	1	1	0	20	54	108
131	6	HS	1	1	0	19	53	108
132	6	HS	1	1	0	19	52	105
133	6	HS	1	1	0	20	52	110
134	6	HS	1	1	0	19	51	109
135	6	HS	1	1	0	18	52	107
136	7	HS	1	1	0	19	52	106
137	7	HS	1	1	0	19	52	105
138	7	HS	1	1	0	18	53	108
139	7	HS	1	1	0	17	53	107
140	7	HS	1	1	0	20	52	106

141	7 HS	1	1	0	17	53	106
142	7 HS	1	1	0	18	53	110
143	7 HS	1	1	0	19	51	108
144	7 HS	1	1	0	18	52	110
145	8 HS	1	1	0	19	52	106
146	8 HS	1	1	0	20	53	105
147	8 HS	1	1	0	19	52	104
148	8 HS	1	1	0	18	51	107
149	8 HS	1	1	0	19	53	107
150	8 HS	1	1	0	19	53	106
151	8 HS	1	1	0	20	54	105
152	8 HS	1	1	0	21	52	107
153	8 HS	1	1	0	22	52	106
154	9 HS	1	1	0	19	51	104
155	9 HS	1	1	0	17	51	107
156	9 HS	1	1	0	18	52	104
157	9 HS	1	1	0	20	51	104
158	9 HS	1	1	0	19	53	109
159	9 HS	1	1	0	19	51	107
160	9 HS	1	1	0	18	51	110
161	9 HS	1	1	0	18	52	108
162	9 HS	1	1	0	19	51	108
163	1 HS+SE	2	1	1	24	54	112
164	1 HS+SE	2	1	1	22	54	111
165	1 HS+SE	2	1	1	25	56	105
166	1 HS+SE	2	1	1	21	57	112
167	1 HS+SE	2	1	1	20	57	110
168	1 HS+SE	2	1	1	22	55	112
169	1 HS+SE	2	1	1	23	57	112
170	1 HS+SE	2	1	1	25	56	106
171	1 HS+SE	2	1	1	24	57	106
172	2 HS+SE	2	1	1	24	54	107
173	2 HS+SE	2	1	1	25	54	107
174	2 HS+SE	2	1	1	23	56	110
175	2 HS+SE	2	1	1	22	55	111
176	2 HS+SE	2	1	1	21	57	112
177	2 HS+SE	2	1	1	25	57	113
178	2 HS+SE	2	1	1	24	54	112
179	2 HS+SE	2	1	1	20	54	110
180	2 HS+SE	2	1	1	25	56	111
181	3 HS+SE	2	1	1	25	54	109
182	3 HS+SE	2	1	1	25	53	107
183	3 HS+SE	2	1	1	21	54	107
184	3 HS+SE	2	1	1	25	54	109
185	3 HS+SE	2	1	1	23	56	110
186	3 HS+SE	2	1	1	22	53	111
187	3 HS+SE	2	1	1	22	53	112

188	3 HS+SE	2	1	1	21	57	110
189	3 HS+SE	2	1	1	21	53	110
190	4 HS+SE	2	1	1	20	53	111
191	4 HS+SE	2	1	1	24	53	112
192	4 HS+SE	2	1	1	25	53	112
193	4 HS+SE	2	1	1	24	55	108
194	4 HS+SE	2	1	1	24	53	109
195	4 HS+SE	2	1	1	22	54	107
196	4 HS+SE	2	1	1	21	55	110
197	4 HS+SE	2	1	1	23	54	112
198	4 HS+SE	2	1	1	20	53	109
199	5 HS+SE	2	1	1	22	54	111
200	5 HS+SE	2	1	1	22	56	109
201	5 HS+SE	2	1	1	21	55	107
202	5 HS+SE	2	1	1	20	54	109
203	5 HS+SE	2	1	1	23	54	110
204	5 HS+SE	2	1	1	23	55	112
205	5 HS+SE	2	1	1	21	53	112
206	5 HS+SE	2	1	1	23	56	110
207	5 HS+SE	2	1	1	22	55	111
208	6 HS+SE	2	1	1	22	55	112
209	6 HS+SE	2	1	1	20	55	110
210	6 HS+SE	2	1	1	21	53	109
211	6 HS+SE	2	1	1	21	56	109
212	6 HS+SE	2	1	1	22	56	112
213	6 HS+SE	2	1	1	24	53	108
214	6 HS+SE	2	1	1	21	54	107
215	6 HS+SE	2	1	1	25	54	111
216	6 HS+SE	2	1	1	22	56	112
217	7 HS+SE	2	1	1	22	57	110
218	7 HS+SE	2	1	1	24	53	109
219	7 HS+SE	2	1	1	23	53	108
220	7 HS+SE	2	1	1	22	54	112
221	7 HS+SE	2	1	1	20	55	110
222	7 HS+SE	2	1	1	20	56	111
223	7 HS+SE	2	1	1	24	55	111
224	7 HS+SE	2	1	1	21	55	112
225	7 HS+SE	2	1	1	21	57	107
226	8 HS+SE	2	1	1	21	53	109
227	8 HS+SE	2	1	1	20	53	107
228	8 HS+SE	2	1	1	23	53	111
229	8 HS+SE	2	1	1	22	54	110
230	8 HS+SE	2	1	1	22	55	107
231	8 HS+SE	2	1	1	21	55	107
232	8 HS+SE	2	1	1	21	56	109
233	8 HS+SE	2	1	1	20	53	108
234	8 HS+SE	2	1	1	23	56	108

235	9	HS+SE	2	1	1	20	54	109
236	9	HS+SE	2	1	1	22	54	110
237	9	HS+SE	2	1	1	21	56	110
238	9	HS+SE	2	1	1	21	54	107
239	9	HS+SE	2	1	1	24	55	109
240	9	HS+SE	2	1	1	20	53	110
241	9	HS+SE	2	1	1	20	53	109
242	9	HS+SE	2	1	1	23	55	107
243	9	HS+SE	2	1	1	22	53	109
244	1	SE	3	0	1	26	62	114
245	1	SE	3	0	1	27	63	117
246	1	SE	3	0	1	27	63	117
247	1	SE	3	0	1	26	64	113
248	1	SE	3	0	1	28	61	115
249	1	SE	3	0	1	26	61	113
250	1	SE	3	0	1	28	63	116
251	1	SE	3	0	1	27	60	117
252	1	SE	3	0	1	26	60	115
253	2	SE	3	0	1	27	59	116
254	2	SE	3	0	1	26	62	114
255	2	SE	3	0	1	28	62	116
256	2	SE	3	0	1	27	63	116
257	2	SE	3	0	1	28	63	115
258	2	SE	3	0	1	26	61	113
259	2	SE	3	0	1	26	59	118
260	2	SE	3	0	1	28	61	116
261	2	SE	3	0	1	26	61	116
262	3	SE	3	0	1	28	63	114
263	3	SE	3	0	1	27	60	117
264	3	SE	3	0	1	27	60	117
265	3	SE	3	0	1	28	61	116
266	3	SE	3	0	1	26	60	117
267	3	SE	3	0	1	28	60	116
268	3	SE	3	0	1	28	61	114
269	3	SE	3	0	1	27	63	117
270	3	SE	3	0	1	27	64	116
271	4	SE	3	0	1	27	62	117
272	4	SE	3	0	1	28	62	118
273	4	SE	3	0	1	26	63	114
274	4	SE	3	0	1	26	64	114
275	4	SE	3	0	1	28	64	116
276	4	SE	3	0	1	27	62	117
277	4	SE	3	0	1	27	62	117
278	4	SE	3	0	1	27	61	118
279	4	SE	3	0	1	28	61	117
280	5	SE	3	0	1	26	62	116
281	5	SE	3	0	1	26	62	114

282	5 SE	3	0	1	28	63	116
283	5 SE	3	0	1	27	63	115
284	5 SE	3	0	1	28	61	117
285	5 SE	3	0	1	27	60	117
286	5 SE	3	0	1	28	60	113
287	5 SE	3	0	1	27	62	117
288	5 SE	3	0	1	27	60	115
289	6 SE	3	0	1	27	60	115
290	6 SE	3	0	1	26	63	114
291	6 SE	3	0	1	28	62	115
292	6 SE	3	0	1	28	60	116
293	6 SE	3	0	1	27	61	117
294	6 SE	3	0	1	27	61	116
295	6 SE	3	0	1	28	62	116
296	6 SE	3	0	1	27	62	116
297	6 SE	3	0	1	27	61	114
298	7 SE	3	0	1	27	61	116
299	7 SE	3	0	1	28	62	117
300	7 SE	3	0	1	27	60	117
301	7 SE	3	0	1	27	60	118
302	7 SE	3	0	1	26	59	117
303	7 SE	3	0	1	28	58	116
304	7 SE	3	0	1	28	59	116
305	7 SE	3	0	1	27	59	117
306	7 SE	3	0	1	28	59	117
307	8 SE	3	0	1	28	62	118
308	8 SE	3	0	1	27	61	117
309	8 SE	3	0	1	27	60	116
310	8 SE	3	0	1	27	62	114
311	8 SE	3	0	1	27	62	116
312	8 SE	3	0	1	28	60	115
313	8 SE	3	0	1	26	62	117
314	8 SE	3	0	1	26	63	117
315	8 SE	3	0	1	28	62	113
316	9 SE	3	0	1	26	60	117
317	9 SE	3	0	1	27	60	115
318	9 SE	3	0	1	27	61	115
319	9 SE	3	0	1	28	61	114
320	9 SE	3	0	1	28	63	115
321	9 SE	3	0	1	27	63	116
322	9 SE	3	0	1	28	60	117
323	9 SE	3	0	1	27	60	116
324	9 SE	3	0	1	27	59	116

EM	FC		
1537.38	2.058047	3164	EN
1616.49	1.922684	3108	EW
1502.75	2.142738	3220	FI
1530.88	2.103365	3220	FC
1511.38	2.074925	3136	
1562.49	2.042893	3192	
1655.08	1.877855	3108	
1497	2.169673	3248	
1563.38	2.05964	3220	
1471.75	2.168847	3192	
1477.75	2.160041	3192	
1556.88	2.068239	3220	
1569.51	2.033756	3192	
1504.62	2.121466	3192	
1477.75	2.178988	3220	
1447	2.186593	3164	
1563.38	2.04173	3192	
1496.75	2.113913	3164	
1510.86	2.149769	3248	
1556.88	2.014285	3136	
1537.38	2.039834	3136	
1471.75	2.168847	3192	
1452.75	2.197212	3192	
1676.64	1.903808	3192	
1655.64	1.944867	3220	
1496.75	2.095206	3136	
1562.86	1.988662	3108	
1616.76	1.957	3164	
1596.51	2.034438	3248	
1530.62	2.067136	3164	
1562.86	2.042409	3192	
1556.88	2.068239	3220	
1627.64	1.978324	3220	
1616.49	1.940006	3136	
1595.97	2.000038	3192	
1530.88	2.066785	3164	
1563.38	2.02382	3164	
1504.62	2.140075	3220	
1452.75	2.158665	3136	
1530.88	2.085075	3192	
1569.51	2.033756	3192	
1556.62	2.086572	3248	
1562.86	2.024494	3164	
1472	2.168478	3192	
1503.25	2.142026	3220	
1556.62	2.032609	3164	

1536.86	2.1134	3248
1447	2.205943	3192
1596.51	1.964285	3136
1589.49	2.025807	3220
1562.86	2.006578	3136
1447	2.186593	3164
1563.38	2.04173	3192
1496.75	2.113913	3164
1562.86	2.042409	3192
1530.88	2.121656	3248
1563.38	2.00591	3136
1504.62	2.140075	3220
1510.86	2.149769	3248
1472	2.149457	3164
1623.51	1.931617	3136
1496.75	2.132621	3192
1562.86	2.042409	3192
1530.88	2.048495	3136
1563.38	2.05964	3220
1471.75	2.130797	3136
1536.86	2.1134	3248
1616.76	1.974319	3192
1627.64	1.961122	3192
1496.75	2.113913	3164
1622.97	1.966765	3192
1530.88	2.158236	3304
1537.38	2.07626	3192
1496.75	2.170035	3248
1622.97	2.001269	3248
1562.76	2.04254	3192
1623.51	2.01785	3276
1530.62	2.158602	3304
1536.86	2.131619	3276
1447	2.263994	3276
1616.49	2.009292	3248
966.72	3.070175	2968
1102.6	2.793397	3080
985.53	3.096811	3052
1157.31	2.588762	2996
915.84	3.210168	2940
938.34	3.103353	2912
985.53	3.011578	2968
1009.09	2.969012	2996
1097.6	2.704082	2968
1037.4	2.860999	2968
1028.09	2.995847	3080
985.72	3.096214	3052

920.34	3.194472	2940
1089.27	2.724761	2968
1062.2	2.820561	2996
933.84	3.208258	2996
971.47	3.026342	2940
898.79	3.364523	3024
1094.31	2.737798	2996
1037.6	2.860447	2968
956.34	3.103499	2968
966.53	3.186657	3080
919.87	3.287421	3024
933.84	3.29821	3080
990.47	2.996557	2968
1037.4	2.834008	2940
1115.31	2.610933	2912
1017.6	2.944182	2996
1042.6	2.873585	2996
1017.6	2.916667	2968
1073.73	2.738119	2940
969.66	3.118619	3024
1009.09	2.969012	2996
881.96	3.333485	2940
1042.6	2.954153	3080
966.53	3.157688	3052
919.98	3.256593	2996
966.72	3.070175	2968
969.66	3.003114	2912
1037.6	2.806476	2912
1094.73	2.736748	2996
1004.53	3.010363	3024
1009.09	2.941264	2968
951.84	3.206421	3052
971.47	3.026342	2940
1004.53	2.898868	2912
1042.2	2.847822	2968
881.96	3.301737	2912
1082.6	2.793275	3024
1004.53	3.010363	3024
990.09	2.969427	2940
1037.6	2.968389	3080
971.47	3.141631	3052
933.84	3.208258	2996
990.47	2.996557	2968
985.53	2.983166	2940
955.98	3.163246	3024
898.96	3.33274	2996
1042.6	2.846729	2968

898.79	3.302217	2968
955.98	3.221825	3080
966.72	3.128103	3024
933.66	3.298845	3080
985.72	3.010997	2968
1062.6	2.766798	2940
985.53	2.954755	2912
919.98	3.256593	2996
1004.72	2.981925	2996
1004.72	2.954057	2968
1082.6	2.715684	2940
1089.27	2.750466	2996
1146.42	2.588929	2968
966.72	3.012248	2912
869.21	3.446808	2996
933.66	3.118908	2912
1022.2	2.848758	2912
1004.72	3.037662	3052
966.53	3.099749	2996
919.98	3.347899	3080
933.84	3.238242	3024
966.53	3.128718	3024
1292.88	2.425592	3136
1190.42	2.610843	3108
1397	2.10451	2940
1199.73	2.613921	3136
1137.4	2.70793	3080
1212.42	2.586562	3136
1308.24	2.397114	3136
1403.25	2.11509	2968
1364.88	2.17455	2968
1298.64	2.307029	2996
1347	2.224202	2996
1290.99	2.385766	3080
1207.14	2.574681	3108
1199.31	2.614837	3136
1422	2.225035	3164
1299.12	2.413942	3136
1077.4	2.858734	3080
1402.75	2.215648	3108
1347	2.265776	3052
1328.25	2.255599	2996
1131.27	2.648351	2996
1352.75	2.256145	3052
1285.24	2.39644	3080
1163.14	2.672077	3108
1163.36	2.69564	3136

1199.73	2.567244	3080
1110.27	2.7741	3080
1062.2	2.926003	3108
1269.12	2.471004	3136
1328.25	2.361001	3136
1316.88	2.296337	3024
1274.64	2.394402	3052
1185.36	2.527502	2996
1152.27	2.672985	3080
1244.53	2.519827	3136
1057.6	2.885779	3052
1190.86	2.609879	3108
1229.14	2.483037	3052
1157.31	2.588762	2996
1077.6	2.83222	3052
1244.99	2.473915	3080
1262.01	2.484925	3136
1115.31	2.811774	3136
1285.24	2.39644	3080
1212.86	2.562538	3108
1207.14	2.597876	3136
1097.6	2.806122	3080
1115.73	2.735429	3052
1173.27	2.601277	3052
1234.42	2.540464	3136
1269.12	2.382753	3024
1136.73	2.63563	2996
1346.75	2.307778	3108
1234.42	2.540464	3136
1251.36	2.461322	3080
1268.88	2.405271	3052
1221.53	2.475584	3024
1185.36	2.64561	3136
1097.4	2.806634	3080
1117.4	2.781457	3108
1322.64	2.349846	3108
1152.48	2.721088	3136
1199.73	2.497229	2996
1110.27	2.748881	3052
1062.2	2.820561	2996
1216.24	2.555417	3108
1190.86	2.586366	3080
1207.14	2.481899	2996
1157.31	2.588762	2996
1173.48	2.600811	3052
1062.6	2.84585	3024
1285.01	2.353289	3024

1082.2	2.820181	3052
1185.36	2.598367	3080
1178.73	2.612982	3080
1131.27	2.648351	2996
1322.64	2.307506	3052
1057.6	2.912254	3080
1062.6	2.8722	3052
1262.01	2.373991	2996
1168.42	2.612074	3052
1608.88	1.983989	3192
1697.49	1.929908	3276
1697.76	1.929601	3276
1667.38	1.897588	3164
1704.36	1.889272	3220
1588.86	1.991365	3164
1760.64	1.844784	3248
1623.51	2.01785	3276
1556.62	2.068584	3220
1595.97	2.035126	3248
1608.88	1.983989	3192
1732.36	1.874899	3248
1703.97	1.906137	3248
1760.64	1.82888	3220
1589.38	1.990713	3164
1530.62	2.158602	3304
1711.08	1.898216	3248
1582.88	2.051956	3248
1767.64	1.805798	3192
1616.49	2.026613	3276
1622.97	2.018522	3276
1704.64	1.905388	3248
1563.38	2.09546	3276
1676.36	1.937531	3248
1711.08	1.865488	3192
1697.76	1.929601	3276
1724.49	1.883455	3248
1676.97	1.953523	3276
1732.64	1.906917	3304
1634.62	1.952747	3192
1660.62	1.922174	3192
1795.08	1.80939	3248
1670.76	1.960784	3276
1677.51	1.952894	3276
1643.49	2.010356	3304
1711.08	1.91458	3276
1608.88	2.018796	3248
1615.38	1.976006	3192

1760.36	1.845077	3248
1703.97	1.889705	3220
1704.64	1.921813	3276
1623.51	2.01785	3276
1676.36	1.887423	3164
1676.97	1.953523	3276
1616.76	1.991638	3220
1623.51	1.983357	3220
1634.62	1.952747	3192
1739.08	1.851554	3220
1676.64	1.937208	3248
1650.51	1.984841	3276
1643.49	1.976282	3248
1739.08	1.867654	3248
1670.76	1.944025	3248
1643.49	1.942208	3192
1643.76	1.975958	3248
1739.64	1.883148	3276
1616.49	2.026613	3276
1622.97	2.035774	3304
1530.88	2.139946	3276
1627.64	1.995527	3248
1648.36	1.970443	3248
1595.97	2.05267	3276
1648.64	1.987092	3276
1739.64	1.899244	3304
1643.49	1.993319	3276
1622.97	2.001269	3248
1670.76	1.910508	3192
1677.51	1.936203	3248
1676.36	1.920828	3220
1614.86	2.028659	3276
1634.88	2.003817	3276
1739.64	1.818767	3164
1556.62	2.10456	3276
1622.97	1.984017	3220
1643.76	1.958923	3220
1711.64	1.864878	3192
1760.36	1.829171	3220
1703.97	1.906137	3248
1676.64	1.953908	3276
1616.49	2.009292	3248
1589.76	2.043076	3248
