# Supplementary material for: Effect of Organic Selenium-Enriched Yeast on Relieving the Deterioration of Layer Performance, Immune Function, and Physiological Indicators Induced by Heat Stress
Source: Front Vet Sci. 2022 Apr 28;9:880790. doi: 10.3389/fvets.2022.880790 (PMC9096893; doi:10.3389/fvets.2022.880790)
Supplement: Supplementary file 2 [file Data_Sheet_2.pdf]

	NO	REPLCT	CODE	GROUP	HS	SY	T cell	B cell
control	1	1	CONT	0	0	0	3.14	2.13
	2	2	CONT	0	0	0	2.56	1.93
	3	3	CONT	0	0	0	2.87	1.87
	4	4	CONT	0	0	0	2.61	2.24
	5	5	CONT	0	0	0	2.95	1.75
	6	6	CONT	0	0	0	3.57	1.83
	7	7	CONT	0	0	0	2.26	2.07
	8	8	CONT	0	0	0	2.57	1.86
	9	9	CONT	0	0	0	2.77	1.96
heat stress	10	1	HS	1	1	0	0.97	0.91
	11	2	HS	1	1	0	0.86	1.05
	12	3	HS	1	1	0	1.34	1.32
	13	4	HS	1	1	0	0.88	1.19
	14	5	HS	1	1	0	1.57	0.85
	15	6	HS	1	1	0	1.63	1.16
	16	7	HS	1	1	0	1.27	1.34
	17	8	HS	1	1	0	1.94	1.25
	18	9	HS	1	1	0	0.87	1.37
HS + sel	19	1	HS+SE	2	1	1	1.91	2.14
	20	2	HS+SE	2	1	1	2.28	1.99
	21	3	HS+SE	2	1	1	2.54	1.81
	22	4	HS+SE	2	1	1	2.33	2.57
	23	5	HS+SE	2	1	1	2.36	2.79
	24	6	HS+SE	2	1	1	2.27	2.15
	25	7	HS+SE	2	1	1	2.65	2.05
	26	8	HS+SE	2	1	1	2.44	1.98
	27	9	HS+SE	2	1	1	2.04	2.39
Sel	28	1	SE	3	0	1	3.61	2.85
	29	2	SE	3	0	1	3.23	2.71
	30	3	SE	3	0	1	2.91	2.34
	31	4	SE	3	0	1	3.86	2.45
	32	5	SE	3	0	1	3.12	2.81
	33	6	SE	3	0	1	2.94	2.89
	34	7	SE	3	0	1	3.21	2.64
	35	8	SE	3	0	1	3.61	2.57
	36	9	SE	3	0	1	3.57	2.69

WBC1000/r	H/L	SRBC titer	villi height	cript depth	villi/cript	IL-1β, ng/m	TNF pg/mL	Corti pg/m
55.3	0.36	8	1950	410	4.75	0.29	94.86	5.44
66.1	0.41	7	1937	380	5.09	0.37	90.23	4.36
49.2	0.27	9	1922	350	5.49	0.17	98.66	5.97
63.4	0.32	8	1881	405	4.64	0.47	87.26	3.95
59.2	0.33	9	1795	400	4.48	0.22	81.65	5.22
67.8	0.29	10	1963	450	4.36	0.19	98.11	4.73
58.3	0.34	8	2015	504	3.99	0.26	85.44	4.69
52.1	0.39	7	2039	491	4.15	0.33	95.36	5.03
65.3	0.37	8	1977	413	4.7	0.24	91.27	4.77
33.1	1.1	6	1729	350	4.94	0.93	178.11	14.86
32.3	0.91	4	1722	333	5.17	0.87	198.14	11.39
40.1	0.78	5	1757	381	4.61	0.81	194.33	13.69
38.2	0.86	4	1805	375	4.81	0.73	150.46	17.24
36.3	0.79	3	1790	400	4.47	0.86	166.71	9.64
29.6	0.92	5	1843	295	6.24	0.79	171.29	16.56
30.7	0.97	6	1659	385	4.31	0.67	200.19	14.19
34.3	0.81	3	1795	399	4.49	0.94	188.37	13.64
40.1	0.87	5	1854	376	4.93	0.88	173.49	12.77
45.7	0.71	7	2045	390	5.24	0.52	140.13	9.26
50.1	0.68	6	1967	409	4.8	0.44	159.17	7.39
40.2	0.59	6	1885	412	4.57	0.39	125.39	8.34
49.6	0.75	7	2075	431	4.81	0.62	139.97	9.69
48.2	0.71	6	1759	370	4.75	0.59	167.09	6.99
40.7	0.63	8	1968	395	4.98	0.37	122.94	12.46
42.3	0.89	6	1790	417	4.29	0.62	144.36	7.29
44.8	0.49	7	1815	379	4.78	0.41	170.66	9.79
49.9	0.62	7	1955	388	5.03	0.53	137.77	8.89
66.6	0.31	8	2250	420	5.35	0.26	93.66	3.66
65.7	0.33	9	2600	450	5.77	0.19	85.39	2.99
60.2	0.36	9	2100	499	4.21	0.29	90.33	4.69
50.8	0.37	10	2150	436	4.93	0.31	77.98	5.33
55.9	0.29	10	2300	545	4.22	0.21	84.67	4.97
70.3	0.27	9	1998	467	4.27	0.16	93.08	3.55
61.8	0.38	8	1956	477	4.11	0.25	87.64	6.49
64.5	0.31	9	1967	513	3.83	0.33	83.55	2.58
70.3	0.35	9	2157	439	4.91	0.18	91.37	4.59

MDA µM/mL
2.01
1.89
1.49
2.36
2.49
2.07
1.98
2.34
2.78
4.35
5.33
6.32
4.12
3.97
4.67
4.99
5.06
3.49
2.56
2.94
2.37
1.97
2.59
2.79
3.15
1.76
2.95
1.6
1.36
1.87
2.16
1.98
1.47
1.66
1.54
1.39
